# Validation of the Spanish Version of the Body Awareness Questionnaire (BAQ) and an Exploration of Its Relationship to Meditation and Embodiment Variables

**DOI:** 10.3390/healthcare13060628

**Published:** 2025-03-14

**Authors:** Laura C. Sánchez-Sánchez, Amanda Klysing, Ingela Steij Stålbrand, Tove Lundberg

**Affiliations:** 1 Department of Personality, Evaluation and Psychological Treatment, Faculty of Psychology, University of Granada, 180071 Granada, Spain; 2 Department of Psychology, Faculty of Social Sciences, Lund University, Allhelgona Kyrkogata 16a, 221 00 Lund, Sweden; amanda.klysing@psy.lu.se (A.K.); ingela.steij_stalbrand@psy.lu.se (I.S.S.); tove.lundberg@psy.lu.se (T.L.)

**Keywords:** surveys and questionnaires, statistical factor analysis, interoception, self-compassion, meditation

## Abstract

**Purposes**: The Body Awareness Questionnaire (BAQ) has been considered the best available measure of body awareness, but it is not currently available in Spanish. **Methods**: To address this shortcoming, a sample of 281 Spanish participants completed a survey with a Spanish version of the BAQ, as well as the Body Appreciation Scale (BAS-2), the Self-Compassion Scale-Short (SCS-S) and the New Sexual Satisfaction Scale-Short (NSSS-S). **Results**: Analysis of the Spanish BAQ showed good reliability: α = 0.82. Positive correlations with the BAS-2 and the SCS-S, and no significant correlation with the NSSS-S, support the convergent and discriminant validity of the Spanish BAQ. In conceptual validity, the Spanish BAQ further successfully discriminated between meditators and non-meditators, showing additional support for the conceptual validity of the measure. Confirmatory Factor Analysis (CFA) supported a two-factor structure, rather than a one-factor or four-factor version, as proposed for the original English version. **Conclusions**: The Spanish version of the BAQ has shown adequate reliability and validity and would be a good scale to continue exploring in clinical Spanish population samples, e.g., in patients with chronic pain, and non-clinical ones, e.g., after interventions in sexuality or sport. It could be an interesting questionnaire to assess outcomes of mindfulness-based interventions.

## 1. Introduction

Body awareness is usually defined as intentionally focusing on and being aware of one’s body sensations [1]. In recent decades there has been a growing research interest in the role of body awareness in relation to health [2,3]. When defined as an ability to recognize subtle body cues, studies have shown that body awareness can be beneficial in managing health conditions, such as different types of long-term pain [4] or congestive heart failure [5], as well as mental health issues [6]. Enhancing body awareness can thus be an important part of interventions promoting well-being [3] and improving sexuality [7]. Drawing on these results, more research needs to be conducted to better understand how body awareness relates to well-being in different population groups and in different cultures. An important aspect of conducting this research is ensuring that there is a valid measurement instrument for body awareness available in multiple languages.

In a review of self-report instruments for body awareness, Mehling et al. [2] concluded that the Body Awareness Questionnaire (BAQ) [8] was the best performing measure. BAQ was developed to measure a person’s sensitivity to non-emotive and normal bodily processes and consists of 18 items. These items were selected to capture “sensitivity to body cycles and rhythms, ability to detect small changes in normal functioning, and ability to anticipate bodily reactions” [8] (p. 802). Even though the BAQ is intended to function as a unidimensional measure, exploratory factor analysis indicated that the items fall into four factors relating to ‘noting responses or changes in body processes’, ‘predicting body reactions’, ‘sleep–wake cycle’, and ‘prediction of onset of illness’ [8]. Various studies have supported the reliability and discriminant as well as convergent validity of the BAQ [2]. For instance, the BAQ discriminates between body awareness and general neuroticism [8] and is positively correlated with measures of self-consciousness [9]. The instrument has been translated into several different languages, such as Swedish [10], Turkish [9] and German [11], among others. However, a Spanish version of the BAQ has not been developed and evaluated.

The current study addresses this lacuna by conducting a translation, psychometric evaluation, and validation of the BAQ in Spanish. To evaluate the discriminant and convergent validity of the translated BAQ, it is important to explore its relationships with associated but distinct constructs. The choice of other variables has been informed by the model of Farb et al. [12], who theorize that interoceptive awareness, e.g., body awareness, forms a foundational layer of interoception, which can influence higher-order constructs. They suggest, for example, that problematic interoceptive processes can lead to a vast array of health problems. For example, awareness of visceral sensations can be beneficial or harmful, depending on how this is interpreted. While contemplative practices may enhance well-being, misinterpretation of interoceptive signals can contribute to panic disorders. Understanding how to skillfully engage with these sensations is crucial for advancing interoceptive training. The representation of the momentary sensory self is key to compassion and insight practices, as well as the foundational mindfulness practices that support them. For this reason, in this particular study, we chose to study body appreciation [13], self-compassion [14], and sexual satisfaction [15]. These were selected as they are three very different constructs, but all related to health and theoretically understood as higher-order processes related to body awareness. As such, including them in the study provides, in addition to evaluations of validity, tentative results on the relationships between body awareness and these constructs. As a validated measure on body awareness in Spanish is missing, this is knowledge urgently needed in a Spanish context in relation to a range of different health-related research areas. As such, an additional aim is to provide a basis from which different researchers can develop future research.

Body appreciation is a central aspect of positive body image. It is conceptually linked to body awareness but remains theoretically distinct. Body awareness emphasizes attentiveness to internal bodily signals, whereas body appreciation focuses on respecting, accepting, and valuing the body [13]. Previous research suggests that awareness of the body can be a foundation for body appreciation [16]. The Body Appreciation Scale-2 (BAS-2) is widely used and validated across diverse populations to measure this construct [13]. Including the BAS-2 allows for an examination of whether the BAQ correlates with body appreciation as expected while maintaining distinctiveness, thereby establishing discriminant validity.

Self-compassion is another construct theoretically linked to body awareness. Like body awareness, self-compassion also requires an element of interoceptive ability. However, self-compassion is also an evaluation of one’s state of being, and involves recognizing one’s needs and responding to them with care [14]. As mentioned above, it is not only important to be aware of the sensations of the present moment, but this attention needs to be compassionate so that it can lead to health care and well-being rather than the opposite. The Self-Compassion Scale—Short Form (SCS-S) has demonstrated robust psychometric properties and is frequently employed in studies exploring interoception-related constructs [17]. By examining the relationship between the BAQ and SCS-S scores, the study can assess whether body awareness is related to self-compassion as hypothesized, without complete overlap.

Finally, the relationship between body awareness and sexual satisfaction is of growing interest in research. Sexual satisfaction, as measured by the New Sexual Satisfaction Scale—Short (NSSS-S) [15], captures an important dimension of well-being [18]. Previous studies have shown that mindfulness practices focusing on body awareness can enhance sexual functioning by reducing psychological barriers and increasing attunement to physical sensations [19]. This suggests a theoretical connection between body awareness and sexual satisfaction, where body awareness may contribute to but remain distinct from sexual satisfaction. Investigating these constructs in tandem allows for a nuanced understanding of their interplay while providing further evidence for the construct validity of the BAQ.

To establish known-groups validity [20], we will further test the ability of the Spanish BAQ to distinguish between two groups expected to differ in body awareness: meditators and non-meditators. These groups were chosen based on previous findings indicating that practicing meditation can increase levels of body awareness through fostering mindfulness [14].

The aim of the present study is to evaluate the psychometric properties of a Spanish version of the BAQ by conducting factor analyses of the structure of the measure. To this objective, a unidimensional factor structure as well as the suggested four-factor structure will be tested through confirmatory factor analyses. The discriminant and convergent validity of the measure will be evaluated through correlations of BAQ scores to other concepts related to self-awareness, specifically body appreciation and self-compassion, while the predictive validity of the translated BAQ will be evaluated through its correlation with sexual satisfaction. Finally, we will estimate the conceptual validity of the translated BAQ through a known-groups comparison: people with and without experience of meditation. As experience of meditation is suggested to increase body awareness [19], this should be reflected in higher BAQ scores among individuals with experience of meditation.

## 2. Materials and Methods

### 2.1. Participants and Procedure

Participants were selected through convenience sampling. Inclusion criteria for the sample were to be of legal age, to have had a formal or informal sexual partner in the last 6 months, not to have been diagnosed with any current disorder, and not to be taking psychotropic or other drugs that could affect their sexuality, as the relationship between BAQ and sexual satisfaction was explored. In total, data were collected from a sample of 281 participants, who completed the entire survey, with a mean age of 25.37 years (SD = 7.59), so they were mainly young adults. In terms of socio-demographic data, of the 281 participants, 182 were women, 96 were men, and 3 had a non-binary gender identity (see Table 1 for more data).

The final survey included the participant information sheet, the informed consent form, demographic questions, and the questionnaires presented below. Demographic questions included age, gender, trans experience, sexual orientation, partner status, length of current relationship, level of education, and employment status. Additionally, experience of practicing meditation was assessed.

The survey was hosted on LimeSurvey, an online survey platform that guarantees confidentiality and data security, and distributed through the social networks Instagram and Facebook and the messaging application WhatsApp.

### 2.2. Instruments

#### 2.2.1. Body Awareness Questionnaire (BAQ)

To create a Spanish version of the BAQ, the original English version [8] was translated into Spanish and back-translated into English by two independent bilingual translators (Spanish and English), following the full process of translation and cultural adaptation of health measurement instruments recommended by Ortiz-Gutiérrez and Cruz-Avelar [21]. Once the translation was obtained, three experts in mindfulness and body awareness with more than five years of expertise were consulted to assess the suitability of the translation (see [22]), using a Likert-type scale from 1 (strongly disagree) to 4 (strongly agree). In addition, a blank space was left for comments on the items. All items received a mean score of ≥2.8 for suitability. The only item to receive an additional comment was item 10, for which one of the experts stated that “being the only reverse item in the scale, participants might understand it worse than the rest”. The translated scale can be found in the Supplementary Materials and consists of 18 items answered on a Likert-type scale ranged from 1 (completely false) to 7 (completely true). The BAQ was developed as a unidimensional measure, but the original version was found to be structured into four overlapping factors: perception of bodily changes (items 1, 4, 10, 13, 14, and 16), prediction of bodily reactions (items 2, 3, 8, 11, 12, 15, and 16), sleep–wake cycle (items 7, 8, 9, 15, 17, and 18), and prediction of illness onset (items 5, 6, 7, and 10). The reliability of the translated BAQ was good α = 0.82, and was further somewhat improved if the reverse-scored item 10 was removed, α = 0.83.

#### 2.2.2. Self-Compassion Scale

The abbreviated Spanish version of the Self-Compassion Scale (SCS) [23] was used to assess general self-compassion. This version of the SCS consists of 12 Likert-type items, where possible responses range from 1 (almost never) to 5 (almost always). It consists of three subscales, Self-Kindness, Common Humanity, and Mindfulness [24], although it can also be considered a unidimensional score. The scale showed good reliability, α = 0.85.

#### 2.2.3. Body Appreciation Scale (BAS-2)

The Spanish version of the Body Appreciation Scale (BAS-2) [25] was used to measure general appreciation of one’s body. The scale consists of 10 items, with Likert-type responses ranging from 1 (never) to 5 (always). The scale showed good reliability, α = 0.93.

#### 2.2.4. New Sexual Satisfaction Scale-Short (NSSS-S)

The Spanish version of the New Sexual Satisfaction Scale-Short (NSSS-S) [15] was used to assess sexual satisfaction. The NSSS-S consists of 12 items measuring overall sexual satisfaction, and responses are made on a Likert-type scale from 1 (not at all satisfied) to 5 (completely satisfied). The scale showed good reliability, α = 0.91.

### 2.3. Data Analysis

The psychometric properties of the translated BAQ were tested using confirmatory factor analysis (CFA). First, we assessed the suitability of the suggested four-factor structure from Shields et al. [8] and then compared this to a single-factor structure, reflecting that the BAQ is intended to be a unidimensional measure. CFAs with maximum likelihood estimation were conducted using the package lavaan [26] in R version 4.1.2 [27]. Comparative Fit Index (CFI), Root Mean Squared Error of Approximation (RMSEA), and the Standardized Root Mean Square Residual (SRMR) were used to assess model fit. CFI > 0.95, RMSEA < 0.06, and SRMR < 0.05 is defined as a good fit [28], while CFI > 0.90, RMSEA < 0.08 [29], and SRMR < 0.08 is defined as an acceptable fit. The internal reliability of the full measure was estimated using Cronbach’s alpha, and validity was additionally investigated through correlations between the BAQ and related measures as well as a known-groups comparison between those with mediation experience and those without.

## 3. Results

To assess the psychometric structure of the translated BAQ, two CFA models were tested. Model 1 consisted of a one-factor model and was created to test whether the BAQ can best be described as a unidimensional measure, as originally suggested by Shields et al. [8]. Model 2 followed the factor structure identified by Shields et al. [8] and included the four aforementioned factors. Before analysis was initiated, we used the response variance and a QQ-plot of the residuals for the scale mean to search for outliers and insincere respondents. Four respondents showed a suspicious answering pattern with little or no variance in responses, including for the reversed item, and their responses to the BAQ were removed before analysis. A total of 277 responses were included in the factor analyses.

To test the factorability of the scale responses, we conducted Bartlett’s test of sphericity [30] and a Kaiser–Meyer–Olkin test [31], as recommended by Dziuban and Shirkey [32]. The Bartlett test was significant and thus indicated that the correlation matrix was suitable for factor analysis, χ^2^(153) = 1180.49, *p* < 0.001. The Kaiser–Meyer–Olkin test showed a ‘meritorious’ sampling adequacy with an MSA of 0.83. However, the MSA for item 10 specifically was only at 0.59, which is defined in Kaiser and Rice [31] as a ‘miserable’ sampling adequacy. For this reason, both factor models were run with and without item 10 to evaluate its impact on the model fit.

The one-factor model showed poor fit, with a CFI of 0.73, an RMSEA of 0.09 (90% CI = 0.08:0.10), and an SRMR of 0.08. Removing item 10 did not improve the model fit; CFI = 0.73, RMSEA = 0.09 (90% CI = 0.08:0.10), SRMR = 0.08. The four-factor model showed a poor fit to the data for CFI (0.80), but acceptable fit for RMSEA and SRMR (RMSEA = 0.08 (90% CI = 0.07:0.09), SRMR = 0.08). Removing item 10 did not improve the model fit: CFI = 0.80, RMSEA = 0.08 (90% CI = 0.07:0.09), SRMR = 0.08. A scaled χ^2^ difference test showed that the four-factor solution had a significantly better fit to the data compared to the one-factor solution, χ^2^ difference(11) = 62.35, *p* < 0.001. However, as the four-factor model still did not reach all the predetermined cut-offs for model fit, we further investigated the factor structure of the translated measure with an exploratory factor analysis (EFA) using the fa function in the psych package [33].

To determine the suggested factor structure of the data, we used the Very Simple Structure test (VSS) [34] for models ranging from 1 to 10 factors. The VSS function included in the psych package provides several goodness-of-fit statistics, including the VSS, the MAP criteria, the BIC, the sample size adjusted BIC, and the RMSEA (see Table 2).

The highest VSS2 score as well as the lowest MAP criterion and BIC occurred for a solution of two factors, while the sample-size-adjusted BIC suggested an optimal solution of five factors. All solutions using more than one factor resulted in an RMSEA value under 0.06, indicating good fit. See Table 2 for fit statistics for different numbers of factors. To provide insights into the structure of the translated measure, we therefore ran two EFAs with either two or five factors using maximum likelihood with robust errors and oblimin rotations. The fit of the two models was compared using χ^2^ difference testing, which showed that the two-factor model had significantly better fit than the five-factor model, χ^2^ (45) = 142.02, *p* < 0.001, indicating that despite the five-factor model showing a lower RMSEA, the two-factor model had better data fit once the complexity of the five-factor model was taken into account. Standardized factor loadings are reported in Table 3. Together, the CFAs and the EFAs indicate that the translated BAQ does not display a one-dimensional nor four-dimensional factor structure, but rather a dual factor structure with items relating to ‘monitoring physical energy levels’ loading on one factor (α = 0.80) and items relating to ‘predicting body responses’ loading on another factor (α = 0.72). The factors correlated positively, r = 0.40, The item relating to not noticing the bodily effects of seasonal rhythms (item 10) loaded poorly on either factor and may not be suitable for inclusion in the scale, either because of its reversed answer format or because of its focus on a more external cyclical process.

To examine the relation of the translated BAQ to related measures, correlations between the Spanish BAQ, the BAS-2, the NSSS-S, and the SCS-S (and its subscales) are reported in Table 3. The BAQ showed significant but weak positive correlations with the BAS-2 and the SCS-S but did not correlate significantly with the NSSS-S. Accordingly, the Spanish BAQ showed convergent validity with the BAS-2 and the SCS-S (as well as its subscales) and divergent validity with the NSSS-S. Descriptive data and correlation coefficients for all measures are presented in Table 4.

We further tested the conceptual validity of the translated BAQ through known-groups comparison between those who meditate and those who do not, two groups assumed to differ in their degree of bodily awareness. In support of the validity of the measure, those who meditated had a significantly higher score on the BAQ (M = 90.71, SD = 15.30) compared to those who did not meditate (M = 82.08, SD = 14.37), as shown by a Welch *t*-test: t(96.48) = 3.99, *p* < 0.001, d = 0.59. Additionally, a second Welch *t*-test was performed to explore a potential difference in BAQ scores based on gender, which showed no significant differences between women and men in the sample, t(197.18) = 0.67, *p* = 0.50.

## 4. Discussion

Firstly, the results are in line with the results found in the English version, showing that the scale presents adequate reliability, with the value of Cronbach’s alpha ranging from 0.80 to 0.88 [5,8]. It is also consistent with the German [11] and Turkish versions [9], as well as the estimated reliability of the Swedish version for a clinical sample [10]. Reliability for the Spanish BAQ was higher than for the Swedish version when used for a non-clinical sample [10].

Regarding the relationship with other scales, the results have confirmed the convergent and discriminant validity of the BAQ, finding a positive and significant correlation with the BAS-2 and SCS scales, but not with the NSSS-S. Because awareness of the body can be thought to be a basis for, but distinct from, appreciation of the body [16], the significant correlation between the BAQ and the BAS-2 supports the convergent and discriminant validity of the translated BAQ. In a similar fashion, both bodily awareness and self-compassion require a degree of introspective ability [14], so the weak but significant correlation between the BAQ and the SCS-S indicates that the translated BAQ is picking up this introspective ability as well as the specific aspects of it related to bodily awareness. Finally, the lack of a significant correlation between the translated BAQ and the NSSS-S supports the discriminant validity of the translated BAQ. These findings converge with previously found correlations between the BAQ and measures such as the Self-Consciousness Inventory and the Body Consciousness Questionnaire, as well as a lack of a correlation with unrelated measures, such as the Rosenberg Self-Esteem Scale [8]. While it is true that the results found are in line with the justification for the selection of these instruments as comparison measures, based on a focus on interoception that can favor health, future studies could include other measures of body connection already validated in Spanish, such as the Scale of Body Connection (SBC) [35]. Although they do not assess exactly the same construct and it has already been pointed out that the BAQ is considered the best scale for assessing body awareness, the inclusion of such scales could increase the correlations found in this study.

With regard to sexuality, although several studies have found changes in body awareness and other variables related to sexual function after Mindfulness-Based Interventions (MBI, e.g., Silverstein et al., [19]), these interventions used scales other than the BAQ, such as the Scale of Body Connection [7,36], or even specific scales of sexual body awareness, such as the Sexual Mindfulness Measure [37]. These scales have been used to compare meditators with non-meditators or to compare the before and after of an MBI applied to sexuality. However, in the present study, only the correlation between the BAQ and the NSSS-S was analyzed directly, but no MBI applied to sexuality was conducted to improve scores on these questionnaires. Correlations between the BAQ and the NSSS-S were also not analyzed with differentiation between meditators and non-meditators. This may have led to the fact that no statistically significant correlations were found between the two scales in the present study. Nevertheless, the BAQ may be appropriate for use in research that aims to improve some aspect of sexuality, e.g., by applying mindfulness meditation (one MBI), taking into account that body awareness may be a mediating or influencing variable in sexuality [38]. In fact, in terms of conceptual validity, statistically significant differences in BAQ scores were obtained between meditators and non-meditators. This result is consistent with those that have found a relationship between meditation and body awareness [39], where body awareness is significantly higher in meditators than in non-meditators [40], as well as in those studies in which an MBI has improved participants’ body awareness [41].

In terms of factor structure, neither the one- or four-dimensional structure identified by Shields et al. [8] showed an optimal fit to the current data. Using exploratory factor analyses, a two-factor structure was instead shown to have the best fit. This is consistent with the Swedish version [10], and concurs with the structure of the German version [11]. This change in the factor structure with respect to the original 1989 English scale, while there was concordance with the factor structure found in later studies, such as the Swedish (from 2013) or the German (from 2018) ones, could point to sociocultural changes. The dissemination among the general population in the West of meditation practices such as MBIs from the 1990s to the present by authors such as Jonh Kabat-Zinn (e.g., Mindfulness-Based Stress Reduction, MBSR) [42] may have had an impact on the way we are aware of our bodily sensations. This could also be the reason for the percentage of participants who meditate within the sample. These socio-cultural changes, with a greater emphasis on mind–body treatments and the fact that the population may have developed a more refined body awareness, may be orienting the factor structure of the scale towards two factors instead of one factor, as was found in the original version several decades ago.

Similar to findings from the English [5] and Swedish versions [10], item 10 showed a weak relationship to the remaining items. This supports the theory that the issue was not with the translation of the item into Spanish, but rather with the content of the item itself. The poorer fit of this item may be due to the fact that it is the only item where the score has to be reversed or because it asks about very general aspects of the influence of seasonal change on the body, which differs considerably from other items. It could therefore be desirable to eliminate item 10 in future versions of the questionnaire.

Regarding the limitations of the study, the sample consisted of a small convenience sample. However, for a first exploratory study of the psychometric properties of this version, it is considered sufficient (e.g., [43]). Nevertheless, the use of the same sample for multiple factor analyses can increase the risk of overfitting and the two-factor structure identified in the current study should therefore be confirmed with additional samples. As it was a convenience sample, the participants were mainly women with a high level of education, which may have influenced the results. However, as there was no significant difference in BAQ score found between women and men, the overrepresentation of women in the sample is unlikely to have substantially influenced the conclusions of the study. The fact that no differences were found between genders may suggest a cultural change in terms of the attention given in our context to the body, where there is increasing attention to health-related factors, such as sports.

On the other hand, the percentage of people who consider themselves bisexual in this sample compared to the general population is striking. This can be considered a strength of this study, as it shows more data from the LGTBIQA+ group than other studies. The timing of the study may also indicate a generational shift in attitudes, labeling, and self-conceptions of sexual orientation. Regarding the representativeness and generalizability of the sample, we are aware that this is only an exploratory study and that these tentative results should be further explored in a Spanish context with larger and more representative samples, to help clarify the reasons for the results found. Although Mehling et al. [2] selected the BAQ as the best scale to assess body awareness, it has not yet been validated in many languages or applied in many studies [44,45]. Therefore, one of the strengths of this study is that it is the first to explore its application in a Spanish sample throughout the Hispanic world. This study can be a starting point for the study of the application of the BAQ in relation to other health variables in clinical samples. For example, it could be applied together with scales such as the Eating Attitudes Test (EAT-40) validated in Spanish (e.g., [46]) to assess changes in people with eating disorders after a psychological intervention (e.g., one MBI). Another strength is the use of expert evaluation of the translation, as well as comparisons between different factor solutions using a diversity of fit measures.

## 5. Conclusions

As a conclusion to the present study, the Spanish version of the BAQ has good reliability and validity (content, construct–convergent and discriminant, and conceptual) in a convenience sample. The utility of the measure should be further explored in studies with clinical (patients with chronic pain, with sexual difficulties, etc.) and non-clinical samples (sport, sexuality, etc.). The factor structure that has obtained the best fit is the dual factor structure, and we may consider the possibility of eliminating item 10 in future applications. Finally, for cross-cultural comparisons, where slightly different versions of the BAQ are used, the unidimensional structure might be optimal, whereas for Spanish-speaking samples, the dual structure would be most appropriate.

## Figures and Tables

**Table 1:** Demographic information for survey sample participants.

	Survey Sample (*N* = 281)
**Gender**	
Women	64.8% (182)
Men	34.2% (96)
Non-binary	3% (3)
**Age**	
Min–Max	17–62
*M* (*SD*)	25.37 (7.59)
**Trans experience**	
Yes	4.63% (13)
No	95.37% (268)
**Relationship length**	
<1 year	67.99%
Min–Max (for >1 year)	1–6 years
*M*_>1 year_ (expressed in months)	32.21
**Sexual orientation**	
Heterosexual	70.8% (199)
Lesbian/gay/homosexual	6% (17)
Bisexual	23.1% (65)
**Occupation**	
Student	54.45% (153)
Employed	34.2% (96)
Self-employed	5% (14)
Unemployed	4.3% (12)
Retired	0.4% (1)
Other	1.8% (5)
**Education level**	
University education	81.9% (230)
Bachelor’s degree	3% (10.7)
Secondary education	2.5% (7)
Other adult education	5% (14)
**Meditation practice**	
Yes	23.1% (65)
No	76.9% (216)

**Table 2:** Fit indices for EFA ranging from 1 to 10 factors.

Number of Factors	Df	VSS2	MAP	BIC	SABIC	RMSEA
1	135	0.00	0.016	−354	74	0.09
2	118	0.64	0.015	−424	−50	0.06
3	102	0.62	0.019	−392	−69	0.05
4	87	0.57	0.025	−345	−69	0.05
5	73	0.54	0.032	−313	−81	0.04
6	60	0.52	0.038	−269	−79	0.02
7	48	0.49	0.047	−221	−69	0.01
8	37	0.44	0.057	−173	−56	0.00
9	27	0.47	0.069	−130	−45	0.00
10	18	0.42	0.087	−89	−32	0.00

*Note.* To allow for cross-loading of items, the VSS for complexity 2 is used.

**Table 3:** Standardized factor loadings and communality score–sum of the squared factor loadings (*h*^2^) for a two-factor EFA.

Item	Factor 1	Factor 2	*h* ^2^
BAQ_15	0.67	−0.15	0.387
BAQ_17	0.65		0.374
BAQ_8	0.63		0.393
BAQ_9	0.59		0.399
BAQ_11	0.53	0.16	0.370
BAQ_16	0.50	0.29	0.449
BAQ_12	0.48		0.245
BAQ_13	0.38	0.10	0.189
BAQ_18	0.30	0.21	0.187
BAQ_6	0.27	0.18	0.150
BAQ_7	0.27	0.18	0.140
BAQ_4		0.67	0.459
BAQ_3		0.62	0.390
BAQ_1		0.60	0.340
BAQ_2		0.51	0.244
BAQ_5	0.11	0.48	0.278
BAQ_14	0.27	0.31	0.235
BAQ_10		0.12	0.027

*Note.* Cutoff for loadings was 0.1.

**Table 4:** Means, standard deviations, and correlations with confidence intervals for BAQ, BAS, NSSS, and SCS-S including subscales.

Variable	M	SD	1	2	3	4	5	6	7
1. BAQ factor 1	52.04	10.37							
2. BAQ factor 2	32.00	7.15	0.45 **						
			[0.35, 0.54]						
3. BAS-2	3.56	0.77	0.32 **	0.12					
			[0.21, 0.42]	[−0.00, 0.23]					
4. NSSS-S	45.22	8.91	0.06	−0.05	0.27 **				
			[−0.06, 0.18]	[−0.17, 0.07]	[0.16, 0.37]				
5. Self-kindness	6.34	1.70	0.28 **	0.03	0.60 **	0.23 **			
			[0.16, 0.38]	[−0.09, 0.15]	[0.51, 0.67]	[0.11, 0.33]			
6. Common humanity	6.18	1.63	0.23 **	0.02	0.48 **	0.23 **	0.59 **		
			[0.11, 0.34]	[−0.10, 0.14]	[0.38, 0.56]	[0.12, 0.34]	[0.51, 0.66]		
7. Mindfulness	6.23	1.76	0.20 **	−0.01	0.48 **	0.17 **	0.68 **	0.61 **	
			[0.08, 0.31]	[−0.13, 0.11]	[0.38, 0.57]	[0.05, 0.28]	[0.61, 0.74]	[0.53, 0.68]	
8. SCS-S	18.75	4.42	0.27 **	0.02	0.60 **	0.24 **	0.87 **	0.84 **	0.89 **
			[0.16, 0.38]	[−0.10, 0.13]	[0.52, 0.67]	[0.12, 0.35]	[0.84, 0.90]	[0.80, 0.87]	[0.86, 0.91]

*Note.* M and SD are used to represent mean and standard deviation, respectively. Values in square brackets indicate the 95% confidence interval for each correlation. ** Indicates *p* < 0.01. BAQ = Body Awareness Questionnaire, BAS-2 = Body Appreciation Scale, NSSS-S = New Sexual Satisfaction Scale-Short form, SCS = Self-Compassion Scale-Short form.

## Data Availability

Data are available upon reasonable request.

## Supplementary Materials

The following supporting information can be downloaded at https://www.mdpi.com/article/10.3390/healthcare13060628/s1, Table S1: Final version of the BAQ items in Spanish and equivalence of items in the English version.

## References

[1] Cioffi D. (1991). Beyond attentional strategies: A cognitive-perceptual model of somatic interpretation. Psychol. Bull..

[2] Mehling W.E., Gopisetty V., Daubenmier J., Price C.J., Hecht F.M., Stewart A. (2009). Body awareness: Construct and self-report measures. PLoS ONE.

[3] Mehling W.E., Wrubel J., Daubenmier J.J., Price C.J., Kerr C.E., Silow T., Gopisetty V., Stewart A.L. (2011). Body awareness: A phenomenological inquiry into the common ground of mind-body therapies. Philos. Ethics Humanit. Med..

[4] Mehling W.E., Hamel K.A., Acree M., Byl N., Hecht F.M. (2005). Randomized controlled trial of breath therapy for patients with chronic low-back pain. Altern. Ther. Health Med..

[5] Baas L.S., Beery T.A., Allen G., Wizer M., Wagoner L.E. (2004). An exploratory study of body awareness in persons with heart failure treated medically or with transplantation. J. Cardiovasc. Nurs..

[6] Krisanaprakornkit T. (2006). Meditation therapy for anxiety disorders. Cochrane Database Syst. Rev..

[7] Sánchez-Sánchez L.C., Valderrama-Rodríguez M.F. (2022). Mindfulness en la salud sexual y bienestar psicológico de profesionales y cuidadores/as de personas en riesgo de exclusión social. Rev. Int. Androl..

[8] Shields S.A., Mallory M.E., Simon A. (1989). The Body Awareness Questionnaire: Reliability and Validity. J. Pers. Assess..

[9] Karaca S., Bayar B. (2021). Turkish version of body awareness questionnaire: Validity and reliability study. Turk. J. Physiother. Rehabil..

[10] Lööf H., Saboonchi F., Henriksson E.W., Lindblad S., Johansson U.B. (2013). THU0460-HPR Psychometric testing of the body awareness questionnaire in an swedish sample of patients diagnosed with rheumatoid arthritis. Ann. Rheum. Dis..

[11] Cramer H., Lauche R., Daubenmier J., Mehling W., Büssing A., Saha F.J., Dobos G., Shields S.A. (2018). Being aware of the painful body: Validation of the German Body Awareness Questionnaire and Body Responsiveness Questionnaire in patients with chronic pain. PLoS ONE.

[12] Farb N., Daubenmier J., Price C.J., Gard T., Kerr C., Dunn B.D., Klein A.C., Paulus M.P., Mehling W.E. (2015). Interoception, contemplative practice, and health. Front. Psychol..

[13] Avalos L., Tylka T.L., Wood-Barcalow N. (2005). The body appreciation scale: Development and psychometric evaluation. Body Image.

[14] Pintado S. (2019). Changes in body awareness and self-compassion in clinical psychology trainees through a mindfulness program. Complement. Ther. Clin. Pract..

[15] Strizzi J., Fernández-Agis I., Alarcón-Rodríguez R., Parrón-Carreño T. (2016). Adaptation of the new sexual satisfaction scale-short form into Spanish. J. Sex Marital. Ther..

[16] Oswald A., Chapman J., Wilson C. (2017). Do interoceptive awareness and interoceptive responsiveness mediate the relationship between body appreciation and intuitive eating in young women?. Appetite.

[17] Raes F., Pommier E., Neff K.D., Gucht D. (2011). Construction and factorial validation of a short form of the Self-Compassion Scale. Clin. Psychol. Psychother..

[18] World Health Organization [WHO] Sexual Health. https://www.who.int/health-topics/sexual-health#tab=tab_1.

[19] Silverstein R.G., Brown A.C.H., Roth H.D., Britton W.B. (2011). Effects of mindfulness training on body awareness to sexual stimuli: Implications for female sexual dysfunction. Psychosom. Med..

[20] Netemeyer R.G., Bearden W.O., Subhash S. (2003). Scaling Procedures; Issues and Applications.

[21] Ortiz-Gutiérrez S., Cruz-Avelar A. (2018). Translation and cross-cultural adaptation of health assessment tools. Actas Dermo Sifiliogr..

[22] Delgado-Rico E., Carretero-Dios H., Ruch W. (2012). Content validity evidences in test development: An applied perspective. Int. J. Clin. Health Psychol..

[23] Garcia-Campayo J., Navarro-Gil M., Andrés E., Montero-Marin J., López-Artal L., Demarzo M.M.P. (2014). Validation of the Spanish versions of the long (26 items) and short (12 items) forms of the Self-Compassion Scale (SCS). Health Qual. Life Outcomes.

[24] Neff K.D. (2003). The development and validation of a scale to measure self-compassion. Self Identity.

[25] Swami V., García A.A., Barron D. (2017). Factor structure and psychometric properties of a Spanish translation of the body appreciation scale-2 (BAS-2). Body Image.

[26] Rosseel Y. (2012). lavaan: An R Package for Structural Equation Modeling. J. Stat. Softw..

[27] R Core Team R: A Language and Environment for Statistical Computing. R Foundation for Statistical Computing. https://www.r-project.org/.

[28] Hu L.T., Bentler P.M. (1999). Cutoff criteria for fit indexes in covariance structure analysis: Conventional criteria versus new alternatives. Struct. Equ. Model..

[29] Little T.D. (2024). Longitudinal Structural Equation Modeling.

[30] Bartlett M.S. (1950). Tests of significance in factor analysis. Br. J. Psychol..

[31] Kaiser H.F., Rice J. (1974). Little jiffy, mark IV. Educ. Psychol. Meas..

[32] Dziuban C.D., Shirkey E.C. (1974). When is a correlation matrix appropriate for factor analysis? Some decision rules. Psychol. Bull..

[33] Revelle W. Psych: Procedures for Personality and Psychological Research, Nothwestern University, Evanston, IL, USA, Version 2.2.9. https://CRAN.R-project.org/package=psych.

[34] Revelle W., Rocklin T. (1979). Very simple structure: An alternative procedure for estimating the optimal number of interpretable factors. Multivar. Behav. Res..

[35] Quezada-Berumen L.D.C., Gonzalez-Ramirez M.T., Cebolla A., Soler J., Garcia-Campayo J. (2014). Body awareness and mindfulness: Validation of the Spanish version of the Scale of Body Connection. Actas Esp. Psiquiatr..

[36] Sánchez-Sánchez L.C., Valderrama-Rodríguez M.F., García-Montes J.M., Petisco-Rodríguez C., Fernández-García R. (2021). Mindfulness in sexual activity, sexual satisfaction and erotic fantasies in a non-clinical sample. Int. J. Environ. Res. Public Health.

[37] Leavitt C.E., Lefkowitz E.S., Waterman E.A. (2019). The role of sexual mindfulness in sexual wellbeing, Relational wellbeing, and self-esteem. J. Sex Marital. Ther..

[38] Seal B.N., Meston C.M. (2007). The impact of body awareness on sexual arousal in women with sexual dysfunction. J. Sex. Med..

[39] Treves I.N., Tello L.Y., Davidson R.J., Goldberg S.B. (2019). The relationship between mindfulness and objective measures of body awareness: A meta-analysis. Sci. Rep..

[40] Cebolla A., Miragall M., Palomo P., Llorens R., Soler J., Demarzo M., García-Campayo J., Baños R.M. (2016). Embodiment and body awareness in meditators. Mindfulness.

[41] Pérez-Peña M., Notermans J., Desmedt O., Van der Gucht K., Philippot P. (2022). Mindfulness-based interventions and body awareness. Brain Sci..

[42] Kabat-Zinn J. (1990). Full Catastrophe Living: Using the Wisdom of Your Body and Mind to Face Stress, Pain, and Illness.

[43] Kyriazos T.A. (2018). Applied psychometrics: Sample size and sample power considerations in factor analysis (EFA, CFA) and SEM in general. Psychology.

[44] Brani O., Hefferon K., Lomas T., Ivtzan I., Painter J. (2014). The impact of body awareness on subjective wellbeing: The role of mindfulness. Int. Body Psychother. J..

[45] Cramer H., Mehling W.E., Saha F.J., Dobos G., Lauche R. (2018). Postural awareness and its relation to pain: Validation of an innovative instrument measuring awareness of body posture in patients with chronic pain. BMC Musculoskelet. Disord..

[46] Petisco-Rodríguez C., Sánchez-Sánchez L.C., Fernández-García R., Sánchez-Sánchez J., García-Montes J.M. (2020). Disordered Eating Attitudes, Anxiety, Self-Esteem and Perfectionism in Young Athletes and Non- Athletes. Int. J. Environ. Res. Public Health.

