# Supplementary material for: Validation of the Spanish Version of the Body Awareness Questionnaire (BAQ) and an Exploration of Its Relationship to Meditation and Embodiment Variables
Source: Healthcare (Basel). 2025 Mar 14;13(6):628. doi: 10.3390/healthcare13060628 (PMC11942399; doi:10.3390/healthcare13060628)
Supplement: Supplementary file 1 [file healthcare-13-00628-s001.zip › healthcare-3425357-supplementary.pdf]

Table S1. Final version of the BAQ items in Spanish and equivalence of items in the English version

English item	Spanish item
1. I notice differences in the way my body reacts to various foods	Noto diferencias en la manera en que mi cuerpo reacciona a los distintos alimentos.
2. I can always tell when I bump myself whether or not it will become a bruise	Cada vez que me doy un golpe sé si me saldrá un moratón o no.
3. I always know when I've exerted myself to the point where I'll be sore the next day	Siempre sé cuándo he hecho un esfuerzo tan grande que al día siguiente estaré dolorido/a.
4. I am always aware of changes in my energy levels when I eat certain foods	Siempre soy consciente de los cambios en mis niveles de energía cuando como ciertas comidas.
5. I know in advance when I'm getting the flu	Sé de antemano cuándo voy a coger la gripe.
6. I know I'm running a fever without taking my temperature	Sé cuándo tengo fiebre sin tener que tomarme la temperatura
7. I can distinguish between tiredness because of hunger and tiredness because of lack of sleep	Puedo distinguir entre cansancio por hambre y cansancio por falta de sueño.
8. I can accurately predict what time of day lack of sleep will catch up with me	Puedo predecir exactamente en qué momento del día me afectará la falta de sueño.
9. I am aware of a cycle in my activity level throughout the day	Soy consciente de un ciclo en mi nivel de actividad a lo largo del día.
10. I *don't* notice seasonal rhythms and cycles in the way my body functions	No noto la influencia de los ritmos y ciclos estacionales en la manera en que mi cuerpo funciona.
11. As soon as I wake up in the morning I know how much energy I'll have during the day	En cuanto me levanto por la mañana, sé cuánta energía tendré durante el día.
12. I can tell when I go to bed how well I will sleep that night	Cuando me acuesto, sé lo bien que voy a dormir esa noche.
13. I notice distinct body reactions when I am fatigued	Noto diferentes reacciones corporales cuando estoy fatigado/a.
14. I notice specific body responses to changes in the weather	Noto respuestas corporales específicas a los cambios del clima.

15. I can predict how much sleep I will need at night in order to wake up refreshed	Puedo predecir cuánto necesito dormir por la noche para despertarme descansado/a.
16. When my exercise habits change, I can predict very accurately how that will affect my energy level	Cuando mis hábitos de ejercicio cambian, puedo predecir con exactitud cómo afectará a mi nivel de energía.
17. There seems to be a "best" time for me to go to sleep at night	Parece que hay un "mejor" momento para irme a dormir por la noche.
18. I notice specific bodily reactions to being overhungry	Noto reacciones corporales específicas cuando estoy muy hambriento/a.
